# “That’s the whole thing about vaping, it’s custom tasty goodness”: a meta-ethnography of young adults’ perceptions and experiences of e-cigarette use

**DOI:** 10.1186/s13011-021-00416-4

**Published:** 2021-11-12

**Authors:** Ananth Ranjit, Grace McCutchan, Katherine Brain, Ria Poole

**Affiliations:** 1 Division of Population Medicine, Cardiff University School of Medicine, Neuadd Meirionnydd, Heath Park, Cardiff, CF14 4YS UK; 2 European Centre for Environment & Human Health, University of Exeter Medical School, Knowledge Spa, Royal Cornwall Hospital, Truro, Cornwall, TR1 3DH UK

**Keywords:** E-cigarettes, Young adults, Harm reduction, Cessation, Perceptions

## Abstract

**Background:**

E-cigarettes are increasing in popularity, particularly among young adults. With public health organisations contesting the possible benefits of e-cigarettes, research is required to explore young adults’ use of e-cigarettes as a smoking cessation and recreational tool. This study examined existing qualitative data to understand how transition into adulthood and issues of identity affect young adults’ perceptions and experiences of e-cigarette use.

**Methods:**

A meta-ethnography was conducted to examine how young adults perceive and use e-cigarettes. Data were synthesised using Noblit and Hare’s (1988) meta-ethnographic approach. Bronfenbrenner’s socio-ecological model (1979) was used to conceptualise themes and map findings.

**Results:**

A total of 34 studies were included in the review. Young adults viewed e-cigarettes as a safer alternative to traditional cigarette smoking and perceived e-cigarettes as an effective cessation tool. Users were able to personalise their e-cigarette use due to the variety of flavours and devices available. E-cigarettes were found to be a sociable tool as they allowed users to align themselves with their peers who used e-cigarettes and facilitated use within smoke-free environments. Young adults demonstrated high levels of self-efficacy with regards to obtaining e-cigarettes from various retailers and were active consumers of e-cigarette marketing.

**Conclusion:**

This meta-ethnography provides an in-depth insight into social norms around e-cigarette use and beliefs that e-cigarettes could be a safer alternative to traditional cigarettes. As young adults increasingly engage with e-cigarettes, there is a need for informed policy decisions regarding appropriate use. Engagement with e-cigarettes is often reflected within social media, so this medium could be a key platform for creating tailored interventions which inform young adults about the appropriate use of these products.

**Supplementary Information:**

The online version contains supplementary material available at 10.1186/s13011-021-00416-4.

## Introduction

Tobacco smoking is a significant public health problem, causing approximately 96,000 deaths per year in the UK [1]. It has been estimated that for every one million cigarette smokers who switch to e-cigarettes, around 6000 deaths could be avoided [1, 2]. However, due to limited evidence about the longer-term public health effects of e-cigarettes, endorsement of e-cigarettes varies. Public Health England promotes e-cigarettes as a safer alternative to traditional cigarette smoking and an effective cessation aid [3]. The World Health Organisation and Public Health Wales advocate for greater restrictive legislation, especially with respect to young adults due to concerns about safety and possible gateway to cigarette smoking [4, 5]. Since the onset of the COVID-19 pandemic, there has been a greater focus on respiratory health and gaining better insight into young adults’ use of e-cigarettes may provide clarity on evolving discussions around regulation [6].

Young adults aged between 16 and 30 years typically encounter great change and instability as they transition into adulthood [7]. This period also reflects their ability to develop autonomy in response to shifting cultural trends [7, 8]. Currently, 4.3% of British young adults aged between 18 and 24 were using e-cigarettes and 3.2 million adult users were recorded [9]. E-cigarette use in United States is three times higher among young adults than older individuals and has been linked to smaller discreet devices which have entered the marketplace [10]. The behavioural similarities to cigarette smoking, perceived health benefits and the recreational element of vaping have introduced e-cigarettes as a potential competitor to traditional tobacco smoking [11].

The possible ‘gateway’ effect of e-cigarette use in young people facilitating the transition to uptake and subsequent traditional cigarette use means there are greater restrictions on advertising and specific devices [12, 13]. The presence of tobacco companies within the e-cigarette industry has raised questions about the proposed use of e-cigarettes as a cessation tool, due to the promotion of fruity flavours and novel devices [14, 15]. Social media is a strong predictor of e-cigarette use and young adults may vicariously learn about e-cigarettes through this medium [16, 17]. Young adults’ offline identity is often intertwined with their online presence and their proclivity to engage in risky behaviours may therefore be influenced by social media [18].

Due to the increasing social acceptability of e-cigarettes, it is important to understand young adults’ perceptions and experiences of e-cigarettes [19]. With an evolving legislative landscape heavily influenced by emotive rhetoric, there is a greater need to appreciate the nuanced conversations provided by qualitative research. This meta-ethnography has conceptualised the discussions occurring among young adults and provides key themes which can be used for the development of policies which are effective and appropriately targeted.

## Methods

The present study used a meta-ethnographic approach (Noblit and Hare, 1988) to identify key themes relating to how transition into adulthood and issues of identity affects young adults’ perceptions and experiences of e-cigarette use [20]. This facilitated the systematic cross-referencing of similar and dissimilar themes relating to young adults’ identity in order to generate a new line of synthesis [20]. This approach was supported by the eMERGe reporting guidelines [21]. The findings were mapped onto Bronfenbrenner’s socio-ecological model, which enables the conceptualisation of human development with respect to the major determinants of health and social wellbeing [22]. This multi-level model assumes that behaviour is influenced by factors at the individual level (e.g. age, sex), the interpersonal level (e.g. relationships with peers/family), the community level (e.g. availability, social norms) and the policy level (e.g. public policy that prohibits/encourage e-cigarette use) [23].

### Systematic search

Key search terms pertaining to ‘young adults’, ‘smoking’ and ‘vaping’ were identified and included in the comprehensive search strategy (Additional file 1). The databases of ASSIA (Applied Social Sciences Index and Abstracts), CINAHL (Cumulative Index to Nursing and Allied Health Literature), Science Citation Index, Social Sciences Citation Index, Embase, MEDLINE, MEDLINE In-Process and PsycINFO databases were systematically searched for English language papers published up to June 2017. Reference lists of included studies were reviewed. Grey literature, such as government reports, were not included. The systematic search was subsequently updated in September 2020.

### Study selection

**Fig. 1:**
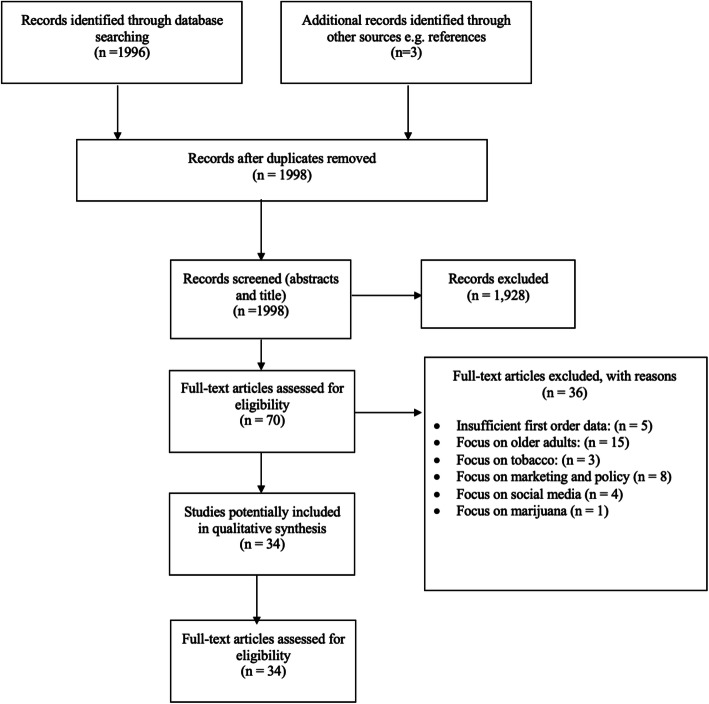
PRISMA flow chart of included studies; Prisma Flow Diagram displays the outcome of the study selection along with exclusion reasons

Two researchers (AR and RP) initially screened titles and abstracts together for the first 10% of studies to reduce risk of bias when assessing potential studies for inclusion. All remaining title and abstracts were reviewed by AR according to the inclusion and exclusion criteria. Where further clarity was required, full texts were reviewed and discussed with RP (see Fig. 1). Study selection was guided by PRISMA [24].

### Inclusion/exclusion criteria

Papers were eligible if research was conducted in high-income countries culturally similar to the UK (for example, USA, Canada, Australia, New Zealand, Western Europe), acknowledging that the legislative framework differs between countries, for example the USA and UK have a more liberal approach to e-cigarette use than Australia and New Zealand. Papers were also eligible if the majority of participants were aged between 16 and 30 years of age. Included studies were required to report experiences and/or perceptions of e-cigarettes and/or vaping and include participant quotes (first order data). Mixed-methods papers were excluded as their limited first order data was insufficient for this meta-ethnographic approach.

### Quality rating

The Critical Appraisals Skills Programme (CASP) qualitative tool was used for study appraisal. Twenty percent of the first set of included papers were independently assessed by a member of the study team using CASP (GM) [25]. There was 75% agreement with quality ratings; one discrepancy was resolved through discussion. Quality ratings were also discussed and agreed with another member of the study team (RP).

### Data extraction

Participant demographics (where available), study design and analysis method were extracted to produce a table of characteristics (see Additional file 2). First order data (participant quotes) and second order data (authors’ narrative interpretations) were extracted along with key study characteristics and coded into NVivo 11 [26].

### Data synthesis

The eMERGe guidelines on how to conduct a meta-ethnography were referred to for step-by-step guidance on data synthesis and interpretation [21]. The first author (AR, a junior doctor with an interest in public health) conducted the synthesis on NVivo. First and second order data were thematically analysed independently to identify key themes. This was an iterative process to ensure a comprehensive understanding of the data. Reflections were logged throughout the process. One other researcher (RP) independently assessed a proportion of the transcripts to reduce bias and confirm findings. Congruent first order themes were grouped, and relevant quotes attached to enable discussions within the study team (RP, GM and KB). Themes derived from second order data were summarised to facilitate study team discussions (RP, GM and KB). First and second order data analyses were reviewed side by side to facilitate idiomatic translation of interpretive themes across both data sets to generate overarching conceptually rich meta-themes (third order data) [20]. This process is sympathetic to reciprocal translation (i.e. the synthesis of similar findings within included papers) and refutational translation (i.e. the synthesis of contradictory findings by identifying disconfirming cases).

Third order constructs were tabulated along with relevant first and second order data. All third order constructs were reviewed individually by AR and RP and then jointly assessed and defined through discussion. At this stage the socio-ecological model was used to categorise third order constructs [23].

## Results

Meta-themes which highlight protective factors and risk factors pertaining to the transition of young adults into and out of e-cigarette use are presented in Additional file 3. The socio-ecological model facilitated the mapping of key themes relating to individual-based decisions on e-cigarette use, the social factors affecting perceptions of these products and regulations which impact accessibility to e-cigarettes.

### 1. Individual use and identity

#### 1.1 E-cigarettes promote positive self-identity

E-cigarettes allowed young adults to project an identity of strength and social standing among peers, whilst also dissociating themselves from the negative stigma associated with traditional cigarettes [27–33].

*“Oh, e-cigarettes are classy, because you can walk around with them. They do not have any vapor that goes around and they look nice.” -* Demographics not recorded [34]

#### 1.2 Expressing individual identity through e-cigarette choice

Many young adults believed that e-cigarettes were an extension of their own identity and e-cigarettes allowed them to reflect their lifestyle choices [27, 28, 31, 35–41]. With a multitude of flavours and e-cigarette designs available, users acknowledged that customisation allowed them to have their own unique experience [28, 30, 33, 36, 37, 39–47]. Many were able to be autonomous in their usage, since they could control the nicotine content of their products [36, 37, 40, 45, 48, 49].

*“Flavours – if I don’t want a menthol or tobacco taste but I still want a bit of nicotine I can go dragon berry or peanut butter gumdrop chew or one of my favourites, which I like is my own mix, it’s pretty good it’s blueberry and marshmallow … that is the whole thing about vaping it’s custom tasty goodness.” -* Demographics not recorded [27]

#### 1.3 E-cigarettes as a playful hobby

Young adults enjoyed the ritual process of maintaining and customising the device and were motivated to learn more about the product in order to maximise their experience [30, 33, 35–37, 39, 40, 44, 45, 47, 50, 51]. Young adults described them as a “toy” and associated use with feeling like a “kid” [37, 42]. The ability to produce large clouds of vapour and perform vape tricks was enticing for young adults [28, 32, 33, 36, 37, 39–41, 44, 46–50, 52].

*“It’s almost like being a kid with a toy. And the way you have to put the oils in some of them, you have to break it down, and take it, and then take the juice and put the little thing and concentrate on it.”* - Male, 25 years old [35]

One study noted that across a 6-month period young adults’ engagement with e-cigarettes was not a fixed pattern due to changing social circumstances, decreasing novelty and shifting public perception [33, 50].

*“I have phases where I’ll go through a lot of the e-juice at the one time and then I’ll just go off it for a wee bit and then just have the occasional one at night … . When I was studying over the prelims, I went through so many bottles because I hate studying and it was just something to do.” -* Male, 16 years old, traditional cigarette user, e-cigarette user [50]

#### 1.4 E-cigarettes a safer alternative to smoking

##### 1.4.1 Successful cessation tool

Participants shared anecdotes of themselves, their friends and family members successfully using e-cigarettes to quit smoking [31, 34, 47, 49, 53]. E-cigarettes allowed users to manage their craving and reduce consumption of traditional cigarettes [27, 29, 30, 33, 35, 39, 49, 50]. Traditional cigarette cessation among young adults was often prompted by key events such as decline in personal fitness, impending parenthood or a disease diagnosis, so e-cigarettes were perceived as a gateway to a more active healthy life [28, 40, 42]. E-cigarettes also allowed users to mimic traditional cigarette cues, habitual behaviours and maintain social rituals [27, 35, 40, 49, 54].

“[ … … .] And I know a friend who started on 10, and it’s a goal for them, it’s like weight-watchers or something. He comes up to me like, “Hey bro! I made it down to 8!” He’s so proud of himself, and now he’s on 4 so … it’s self-rewarding, psychologically.” - Male, 25 years old [35]

However, the behavioural similarities between smoking and vaping were a barrier to smoking cessation for some young adults [30, 35, 41, 44, 47, 53]. The strong relationship between alcohol consumption and use of traditional cigarette caused some young adult e-cigarette users to relapse in certain settings [42, 47].

*“I tried to stop* [smoking] *with a vapouriser but it only went so far … It doesn’t work when you out drinking.”* - Male, 19 years old, traditional cigarette user, ex-e-cigarette user [42]

##### *1.4.2* Fewer chemicals present

Young adults used traditional cigarettes as reference to convey the lack of harm associated with e-cigarettes and a perceived lack of chemicals further supported this [28, 33, 36, 39, 40, 46, 48–52, 54–57]. The absence of abrasive smoke and the physiological benefits contributed to of the perception that e-cigarettes were a healthier alternative to traditional smoking [28, 29, 32, 35, 36, 39, 43, 46, 50, 51, 53, 54, 56, 58].

*“I felt a lot more energetic,* [using e-cigarettes] *a lot more healthy, less out of breath like I can go running again you know versus what I did when I was smoking I mean I couldn’t do it cause I couldn’t breathe.”* - 25 years old, female, ex-traditional cigarette user [28]

#### 1.5 Excessive use and addiction to e-cigarettes

The perceived ability to use e-cigarettes in smoke free environments and the lack of natural “end point” (where a traditional cigarette burns to its end) led users to feel they were excessively consuming nicotine [37, 42, 43, 47, 48, 50, 52, 59]. Young adults acknowledged potential risks such as nicotine dependency due to the inability to quantify consumption, psychological addiction and the addictive appeal of flavourings [32, 33, 37, 43, 45, 48, 52, 58, 60].

*“I know people who, you know, like, will hit their vape once. And I know people who don’t breathe oxygen right? Like, they only use their vape.” -* Male, 23 years old, ex-e-cigarette user, traditional cigarette user [43]

Due to the novelty of the product and inconsistent information provided by health institutions, users were perceived by authors to not fully comprehend the underlying risk of addiction [32, 33, 37, 40, 45, 50, 60, 61]. Conversely, an analysis of first order data showed that young people were adapting to this issue by managing nicotine consumption through moderating time spent on the device and creating their own endpoint [42, 43].

*“You kinda reach a natural end of … ‘right, I’ve been using this for four minutes, that’s like a fag, I’m going to put this away now,’ so it was weird to see someone just sitting constantly* [vaping]*.”* - Female ,22 years old, traditional cigarette user, tried e-cigarettes [42]

#### 1.6 E-cigarettes a risk factor for subsequent tobacco use

Although the leading perceptions of e-cigarettes related to harm reduction and use as a novel product, a minority perceived e-cigarettes as a ‘gateway’ to transition to traditional cigarettes. This was due to the potential ability to become habituated to the similar gestures and sensation between products [31, 33, 44, 45, 47, 53, 61]. Social pressures and inherent proclivity to challenge social norms through risky behaviours were suggested as reasons for the transition to traditional cigarettes [61]. Authors noted that e-cigarette use within smoke free environments may potentially renormalise traditional cigarette use and contribute to the diminishing awareness of the dangers of traditional cigarettes [31, 33, 36, 52, 60, 61].

*“The electronic cigarette can make the gesture a commonplace, one will lose track of the danger of smoking by starting with the [electronic cigarette] just for the taste* [ … ] *and after why not pass on to [traditional cigarettes] which is the following step.” -* Male, 19 years old, traditional cigarette user [61]

### 2. Social use and identity

#### 2.1 E-cigarettes provide social status and group acceptance

E-cigarettes were perceived as fashionable and allowed young adults to align themselves with their peers who also used e-cigarettes [28, 30–33, 36–38, 40, 41, 43, 44, 46, 49, 50, 55, 56]. Users gained social capital by competing with their peers through performing vape tricks and demonstrated their superior engagement with e-cigarette culture through their customised devices [27, 31, 36, 37, 41, 44]. Social media and picture messaging apps such as Snapchat allowed users to show off their e-cigarette use to their friends [28, 32, 40, 48, 50].

*“ … … . breaking the ice in terms of conversation, you have something that you all have in common. You can talk about your different flavours, the brand. There is a history and a commonality between other people.”* - Male [36]

#### 2.2 Relative acceptability of e-cigarettes compared to traditional cigarettes

The visibility, accessibility, and freedom to use e-cigarettes within public spaces prohibited for smoking was a key motivation for exclusively using e-cigarettes [31, 33–35, 37, 43, 44, 46, 48, 52, 56]. Young adult e-cigarette users perceived themselves to be more conscientious than traditional cigarette users within a public space due to the perceived absence of harm from second-hand vapour, the lack of offensive smells and believe that they are better for the environment [29–31, 33, 35, 41, 43, 46, 48, 51, 57, 60].

*“ … when you are sitting next to a person that doesn’t smoke like at the bus stop and you are vaping, they don’t get up and move. When you are smoking they get up and move.” -* Demographics not recorded [27]

#### 2.3 Context-dependent e-cigarette use

Young adults who continued to use traditional cigarettes chose to dual use e-cigarettes to manage their nicotine cravings within smoke free environments and also maintain their perception of professionalism (for example, not taking cigarette breaks at work or smelling of tobacco smoke) [27, 32, 35, 37, 38, 48, 52, 55]. Some dual-users commented on the pressures to switch to traditional cigarettes when in certain social situations, such as at parties or among other cigarette smokers [27, 37]. A small number of young adults noted that they utilised traditional cigarettes when particularly stressed or desired greater nicotine stimulation [27, 37].

*“ … being on 24 hour on call last thing you want to do is show up smelling like an ash tray.” -* Demographics not recorded [27]

#### 2.4 Negative stereotypes of e-cigarette users

Some young adults (non-vapers) perceived e-cigarette users to have negative personality traits such as being “stuck up” and were stigmatised as addicts [27, 29, 30, 36, 42, 44]. The social undesirability attached to e-cigarettes by some young adults was due to them perceiving use as an attempt at being cool and as an outdated ‘fad [29–31, 33, 41, 42, 47, 50].’ Those who had taken up vaping without prior experience with traditional cigarettes were criticised by their peers, since they perceived them as solely a cessation tool [31, 35, 38, 60, 61].

*‘People just kind of mess with you a bit when you’ve got it, people can undermine you a bit and say, “Ha, what a gimp he’s vaping, you know.” -* Male, 16 years old, e-cigarette user, traditional cigarette user [50]

Young adults were concerned about e-cigarette users being stigmatised similarly to tobacco users [47, 51]. The critical discourse around e-cigarettes had forced some individuals to use their products within private spaces [41, 42, 49]. Negative stereotypes existed within e-cigarette communities as hierarchies of device were perceived among vapers; with use of larger devices being seen as ostentatious e.g. Box Mods, whilst smaller devices, especially the brand ‘Juul,’ were seen as more acceptable and discreet [41, 43].

*“So I went and bought an e-cigarette* [larger device], *and the I felt really awkward using e-cigarettes cause they’re douche, and so I got a JUUL because JUULs for some reason aren’t douchey.”* - Male, 21 years old, e-cigarette user [43]

Some young adults perceived themselves to have a healthy lifestyle as they had full control over their traditional cigarette use and able to quit at any time. E-cigarette were seen as disrupting this carefully crafted identity and use was equated to accepting that they had an addiction [42].

*“I’m not addicted to cigarettes. I can smoke for, say, like a year, like consistently, every have a fag … I don’t get addicted.”* - Female, 17 years old, traditional cigarette smoker, ever-vaped [42]

#### 2.5 School and family contexts supporting e-cigarette use

Many families were felt to be supportive of young adults if they used e-cigarettes as a smoking cessation tool [30, 35, 44, 54, 55]. Participants reported that family members had bought e-cigarettes for them and vaping had become a shared experience [27, 30, 31, 35, 36, 44, 57]. Teachers were seen to be tolerant and supportive of e-cigarette use since they were cautious to discourage a cessation tool [31, 44, 46]. E-cigarette use in front of children was seen as acceptable by some users [47, 51].

*“My mother* [would approve of e-cigarette use]. *She would rather me use an e-cigarette than conventional cigarettes, that’s for sure. People that care about me would rather me smoke an e-cig than smoke conventional cigarettes.” -* Female, e-cigarette user [29]

### 3. E-cigarette marketing and availability

#### 3.1 E-cigarettes a superior, long-term cessation tool

Of the young adults who had used alternative nicotine replacement products, e-cigarettes were perceived to be superior long-term cessation tools as they satisfied cravings and provided a better experience [27, 35, 36, 40–42, 45, 49, 54, 55]. Flavours of e-cigarettes could not be matched by traditional cigarettes and the variety offered by e-cigarettes contributed to continuing cessation [33, 41, 43, 45, 47]. However, users conveyed the importance of intrinsic motivation alongside e-cigarettes use in order to successfully maintain smoking abstinence [42].

*“With e-cigarettes you still get to blow out smoke. Because I tried using that Nicorette inhaler, and it just … Ugh … It just didn’t stick with me. I guess because I’m not blowing out smoke, so it felt like I’m not doing anything. And the nicotine gum, that stuff kind of bites the back of your throat, and I don’t really like that feeling.” - Male, 26 years old* [35]

#### 3.2 E-cigarettes easy to obtain

Young adults exhibited high levels of self-efficacy with regards to obtaining e-cigarettes, since various retailers sold them and for those underage, they found few barriers to purchasing them, as it was at the sellers’ discretion to ask for proof of age [29, 31, 32, 35, 36, 44, 52, 57, 58]. If they were unable to purchase e-cigarettes, young adults commented on the reliability of asking proxies, such as strangers or family members, to purchase them on their behalf [31, 44]. E-cigarettes varied in cost and quality; however, users commented on certain e-cigarette products as being cheaper than traditional cigarettes [31, 36, 48, 51, 57]. Those who bought expensive products justified their use by the cost saving accrued over time in comparison to purchasing traditional cigarettes [31, 35, 36, 42, 48, 51].

*“I actually wanted to get it because it was cheaper than smoking cigarettes, because I was smoking like a pack, a pack and a half per day. So it was a lot cheaper than purchasing the cigarettes, which were like $8, if you’re lucky. I mean every pack.” - Male, 25 years old* [35]

#### 3.3 A novel product which appeals to young people

E-cigarettes were noted by young adults to have surpassed outdated traditional cigarettes in terms of fashion and function, since they were a commodity that was inherently youth orientated by design and reflected their intimate relationship with technology [31–33, 37, 40, 41, 43–45, 47, 48, 52, 53, 55, 57, 58]. Expeditious consumption and discreet use were noted by participants across several studies as key features of e-cigarettes [30–33, 35–37, 40, 43, 44, 46, 48, 56, 61]. E-cigarette users preferred vaping to smoking as they saw them as being designed to be more aesthetically pleasing and did not give users “yellow teeth” or produce an offensive smell [35–37, 43, 53].

*“I can do it while studying in the library, upstairs and no one would really know.” -* Demographics not recorded [30]

#### 3.4 E-cigarette companies aggressively targeting young adults

##### 3.4.1 E-cigarette marketing

Exposure to e-cigarettes was noted as being due to the increasing visibility and glamorisation of e-cigarettes within popular press, social media and through celebrity endorsements [29, 34, 48, 58, 60]. Strategic marketing practices such as live demonstrations and free samples were discussed within the first order data (direct quotes) but less noted within second order data (authors’ narrative) [52, 57, 58, 60]. Young adults also displayed brand awareness when prompted by researchers [2, 22, 23]. Marketing claims by e-cigarettes companies were noted to be very persuasive, as they portrayed their products as safe, novel and a healthier alternative to smoking [44, 48, 52, 54, 58].

*“I think the more ads they put up, the more inclined younger people are to try it. Especially if they are flavoured, it’d be interesting to try them.” Female, 17 years old* [58]

##### 3.4.2 Support for stricter regulation on e-cigarettes

Support for e-cigarette regulation was advised with respect to unknown risks of e-cigarettes, age of sale restrictions and reducing the appeal of e-cigarettes to non-smokers [31, 60]. Some participants commented on the restriction of use in public places, where children are present, as it could increase their desire to experiment with these products [31, 41, 60]. Participants supported the grouping of e-cigarette regulation with tobacco legislation as they are both nicotine containing products [29, 31, 51, 60]. Marketing messages were critiqued, and some recommended that they should highlight their sole use for smoking cessation and provide more health information [31, 58]. Individuals expressed concerns about the presence of tobacco companies within e-cigarette marketing and their influence in producing a new generation of users addicted to nicotine [47].

*“The same rules should apply because they’re the same thing aren’t they. They’ve both got nicotine in them.” - Male, 17 years old, e-cigarette user and traditional cigarette user* [31]

#### 3.5  E-cigarettes perceived as a harmful and risky product

E-cigarettes were seen as unsafe by some young adults because they were thought to contain harmful chemicals and pose health risks [32, 38, 40, 45, 57]. Some participants perceived e-cigarettes to be as harmful as traditional cigarettes [32, 38, 42, 50, 58]. A small number of young adults had experienced physiological effects after heavy usage, such as dryness of the throat and a “nicotine hangover” [37, 47, 48, 51]. Whilst dangerous incidents from malfunctioning products were discussed, users commented that product malfunction may be due to user complacency (for example, users not maintaining their e-cigarettes properly) [31, 37].

*“Nothing is really good for you when you inhale it, doesn’t matter what. But these chemicals, putting them into your lungs, it’s still not good for you regardless of what it is, and I’m aware of that, but it’s a self-conscious choice that everyone has to make.” - Female, 19 years old* [37]

#### 3.6 Concern and confusion regarding e-cigarette contents and safety

The perceived lack of research on long-term risks of e-cigarette use affected young adults’ views on the safety profile of e-cigarettes [28, 29, 31–34, 37, 38, 40, 41, 44, 47, 48, 53, 55, 58, 60]. Young adults were obtaining information and developing their views about e-cigarettes from informal channels, such as peers, family, the internet and social media [31, 32, 39, 51]. Young adults recognised the need for reputably sourced information on the health implications of e-cigarettes and their use as a cessation tool [28, 31, 33, 41, 47, 51, 55, 58, 61].

*“I don’t know if they’re any better for you than cigarettes because I feel like there’s a lot of mystery behind them, but I hope [they are better for you than cigarettes].” - Demographics not recorded* [55]

#### 3.7 Indifference about potential harm

Whilst generally unreported within first order data, many authors reflected that the potential health risks did not hinder participants’ choice to use e-cigarettes [27, 31, 33, 36, 51, 52].

*F4: “In the shop I went to in the market, there was side effects on erm the thing it was just like drowsiness and all that. It wasn’t anything major.”*

*F1: “So you still thought you’d buy that?”*

*F4: “Yeah.”*

*F1: “Even though it said it can cause that?”*

*F4: “It’s not anything, it’s nothing major … ”*

*F1: “It’s minor, but it’s still a side effect … ”*

*F4: “If it makes you a bit sleepy than that’s fine, it’s not like a proper drug.”*

-F1: Female, 16 years old, non-e-cigarette user and non-traditional cigarette user

- F4: Female, 16 years old, e-cigarette user and traditional cigarette user [31]

## Discussion

### Summary

This meta-ethnography provides clarity on young adults’ multi-faceted engagement with e-cigarettes and the tailoring of their use to express their individuality. E-cigarettes were viewed as a harm reduction tool, as they were perceived to be safer than traditional cigarettes. E-cigarettes facilitated social cohesion among peers through recreational use. Dual use of e-cigarettes and traditional cigarettes allowed users to maintain their nicotine consumption within smoke-free environments and facilitated a positive identity within the workforce. Young people were aware of the lack of e-cigarettes’ natural end point compared to that of traditional cigarettes and were concerned about nicotine dependency due to excessive vaping. Young adults faced criticism for their use of e-cigarettes and those who used them purely recreationally received particular negative attention. Youth orientated marketing strategies were recognised by young adults as they saw an increasing presence of e-cigarettes on social media and the use of celebrity endorsements. The marketing of e-cigarettes as a sleek device and with a multitude of flavours available led participants to perceive e-cigarettes as an inherently youth orientated tool.

### Social trajectory of e-cigarettes

Young adults reported specific social uses and features of e-cigarettes, which distinguished them from traditional cigarettes. The social trajectory of e-cigarettes away from being a cessation tool was similarly observed in one third of a sample of young adults in California State University, who were non-smokers and used e-cigarettes as a recreational tool due to perceiving them as “trendy” [62]. This shift has been reflected in e-cigarette advertising, with advertising highlighting social acceptability, youth appeal and primarily being placed on online platforms with a large youth audience [63]. Participants were eager to discuss exciting flavours with their peers and a study examining e-cigarette use in youths identified that 81.5% of users attributed initiation of e-cigarettes to flavouring [64]. A UK study examining changes in e-cigarette use during the COVID-19 pandemic found that a large proportion of current users had increased their consumption due to boredom and those who had were typically younger [65]. As young adults have considerably struggled with mental health during this pandemic, the use of e-cigarettes within the context of social isolation, boredom and stress needs to be further explored [66].

### Personalisation of e-cigarettes

As demonstrated by this meta-ethnography, users of e-cigarettes were able to find their own niche within this market, as some were particularly interested in small, discreet products whilst others modified their devices in the pursuit of producing the largest cloud of vapour. The shared experience of e-cigarettes within the young adult and adult market is demonstrated by their customisation, as adults over 30 were found to be equally appreciative of their autonomy over the products and the hobbyist aspect [67]. Social media sites are an outlet for users to showcase their modifications and share information, and regardless of the plethora of devices available, the product ‘JUUL’ appeared to dominate the market [68, 69]. Young adults personalised their use of this discreet device through different flavouring options and were keen to align themselves with this product which has become popular through social media channels and celebrity endorsements [43]. Through using specific terminology such as ‘Juuling’ to describe their use, young adults were making a conscious effort to define their use of e-cigarettes. The reasons for using e-cigarettes are multifaceted and e-cigarette manufacturers are acutely aware of this, as they increasingly develop novel products and advertise their ability to provide consumer choice [70]. As personalisation becomes an increasing point of discussion and feature of young adults’ use, it is important to further examine how the diverse range of products available impact long term use of e-cigarettes.

### Clinical implications

A lack of consistent public health messaging was noted among participants, and this is potentially detrimental as it may lead to young adults dismissing health information regarding e-cigarettes and developing misinformed views. Our meta-ethnography enables policy makers to design and implement effective public health interventions and policy at multiple levels. Social channels such as peers, family and social media were identified as primary points of information but many young adults recognised a need to obtain reputably sourced information. Social media has been recognised as an important health promotion tool since young adults are commonly hard to reach, prolific users of this medium and social media has been implicated in e-cigarette initiation [16, 71, 72]. Social marketing, which has been used to effectively promote healthy behaviour, could utilise social media to encourage e-cigarette use as an aid to quitting in young adults [73].

### Strengths and limitations

Reference to the eMERGe guidelines ensured a comprehensive meta-ethnographic approach [21]. The use of the CASP quality appraisal tool facilitated the quality assessments and ratings of the included papers, and the study team decided to include papers rated as fair because those papers provided sufficiently rich first and second order data. Our study is complemented by a recent meta-ethnography by Smith et al., which explored the social element of e-cigarettes and its role in perceived harm reduction [74]. A strength of the meta-ethnography methodology is that a wide range of perspectives such as primary research participants, primary researchers and secondary researchers were included to generate rich themes regarding the emerging phenomena. To reduce individual researcher bias, regular interpretive discussions and analyses took place within the study team. ‘Juul’ a pod type e-cigarette is a dominant product within the e-cigarette landscape as evidenced by its use, known as ‘Juuling,’ becoming a popular turn of phrase [75]. Its relatively recent presence and impact on young adult use was not fully appreciated in available research at the time of this current meta-ethnography. Further qualitative research is needed to understand how this particular product as well as other pod-based e-cigarettes have changed perspectives among young adults. A potential limitation was the poor reporting of reflexivity across papers. The Covid-19 pandemic has had a global impact on public health and subsequently the timing of this meta-ethnography cannot account for this [76]

### Future research

Most qualitative studies of young adults’ e-cigarette use are US based, and therefore future research is needed within a UK setting. The UK policy framework for health and social care advocates for representative diversity within research, and so further researched is needed to understand perceptions of e-cigarette use among minoritised, racial and cultural groups [77, 78]. Additional primary research may explore young adults’ patterns of e-cigarette use, especially in response to changing legislation and differing country-specific health stances on e-cigarettes.

## Conclusions

This present study provides rich insights into young adults’ perceptions and experiences of e-cigarette use. Whilst the social aspects of e-cigarettes and the ability to personalise their use have considerable appeal, young adult users are at a crossroads due to shifting social norms and receiving conflicting health information. Further high-quality research is needed to ensure that decisions regarding e-cigarette regulation and marketing are evidence-based, which may in turn influence the appropriate use of e-cigarettes among young adults.

## Supplementary Information

**Additional file 1.** List of search terms.

**Additional file 2.** Table of Characteristics of included studies - Extraction of key information from primary data sources such as demographics and data collections methods.

**Additional file 3: Table S1.** Summary of 3rd order constructs and participant quotes.

## Data Availability

The datasets supporting the conclusions of this article are included within the article (and its additional files). The table of characteristics, summary of third order constructs, participants’ quotes and list of search terms are included as additional files.

## Abbreviations

ASSIA: Applied Social Sciences Index and Abstracts
CINAHL: Cumulative Index to Nursing and Allied Health Literature
PRISMA: Preferred Reporting Items for Systematic Reviews and Meta-Analyses
CASP: Critical Appraisals Skills Programme
