# Supplementary material for: “That’s the whole thing about vaping, it’s custom tasty goodness”: a meta-ethnography of young adults’ perceptions and experiences of e-cigarette use
Source: Subst Abuse Treat Prev Policy. 2021 Nov 12;16:85. doi: 10.1186/s13011-021-00416-4 (PMC8586839; doi:10.1186/s13011-021-00416-4)
Supplement: Supplementary file 1 — Additional file 1. List of search terms. [file 13011_2021_416_MOESM1_ESM.pdf]

Additional File 1: Table of Search Terms

#	Searches	Results
1	(teen or teens or teenage*).tw.	23946
2	adolescen*.tw.	199905
3	(youth or youths).tw.	47651
4	((young adj (person* or people or adult*)) or early adult*).tw.	91043
5	(student or students).tw.	198273
6	adolescent/	1851152
7	or/1-6	2072789
8	"Tobacco use"/	811
9	*Smoking/	73289
10	"Tobacco Use Disorder"/px [Psychology]	2640
11	Tobacco products/	2152
12	Vaping/	39
13	(smoke or smoking or smoker or smokers).tw.	211434
14	(vape or vaping or vaper or vapers).tw.	159
15	(cigarette* or cigar or cigars or cigarillo*).tw.	56939
16	("tobacco use" or "nicotine use").tw.	12226
17	or/8-16	231279
18	Qualitative research/ or Phenomenology/ or Focus groups/	52080
19	(qualitative or ethnograph* or grounded theory).tw.	152937
20	(("semi-structured" or semistructured or unstructured or informal or "in-depth" or indepth or "face-to-face" or structured or guide) and (interview* or discussion*)).tw.	87378
21	focus group*.tw.	28344
22	interviews as topic/	53021
23	((audio-recorded or transcribed) and (interview* or survey* or questionnaire*)).tw.	8543
24	(themes adj3 (identif* or explor*)).tw.	8772
25	or/18-24	266415
26	(identit* or reflecti* or meaning* or attitude* or perception* or perceiving*).tw.	584308
27	(self adj (concept or identit* or identif* or labe*)).tw.	7955
28	Attitude/	44341
29	Self concept/	52545
30	Social perception/	20535
31	social identification/	8018
32	or/26-31	661280
33	7 and 17 and 25 and 32	609
34	limit 33 to (english language and yr="1998 -Current")	555
