# Supplementary material for: “That’s the whole thing about vaping, it’s custom tasty goodness”: a meta-ethnography of young adults’ perceptions and experiences of e-cigarette use
Source: Subst Abuse Treat Prev Policy. 2021 Nov 12;16:85. doi: 10.1186/s13011-021-00416-4 (PMC8586839; doi:10.1186/s13011-021-00416-4)
Supplement: Supplementary file 2 — Additional file 2. Table of Characteristics of included studies - Extraction of key information from primary data sources such as demographics and data collections methods. [file 13011_2021_416_MOESM2_ESM.pdf]

Table of Characteristics of included studies

Paper Number	Authors, Date of Publication, Country	Population Age, gender, cultural identification, recruited smoking/vaping status	Recruitment and Data Collection methods	Analysis	Quality Rating
1	Akre et al (1)<br>2016<br>Lausanne, Switzerland	N=42<br>Age:16-26<br>Female:19<br>Male: 23<br><br>E-cigarette user: 74%<br>Dual User: 31%	Participants recruited through social media, university sites and snowball methods<br>Topic guide developed from previous qualitative study on substance use and a literature search<br>8 Focus groups stratified by consumption type<br>Questions centred around:<br>• Consumption<br>• Reasons to use or not to use e-cigarettes<br>• Difference between e-cigarettes and traditional cigarettes<br>• Thoughts on "gate-way effect"	Thematic analysis (2)	Good
2	Antin et al (3)<br>2020<br>San Francisco, USA	N=49<br>Age:15-25<br><br>Female:24<br>Male: 25<br><br>38% White<br>62% Other<br>100%: Ever use of e-cigarette	Participants recruited through social media, on street recruitment methods and snowball methods<br>Semi-structured interviews<br>No mention how topic guide was developed<br>Topic guide centred around:<br>• Participant's history of nicotine therapy (NT) use<br>• Pathway into and current practices of vaping and smoking<br>• Perceptions of vaping and smoking	Thematic analysis (4)	Good
3	Camenga et al (5)<br>2015<br>Public University and Public College,	N=127<br>Age: 13-22<br>Female:61<br>Male:66<br><br>56.7% non-Hispanic White	Purposive sampling of smokers and non-smokers within colleges and high school groups<br>18 Focus groups stratified by gender and smoking status<br>Semi-structured qualitative guides developed from pilot focus group containing high school and college students<br>Questions centred around:<br>• Motivations to use e-cigarettes	Framework analysis (6)	Good

	New Haven County, USA	20.5% non-Hispanic Black<br>10.2% Hispanic White,<br>3.9% Asian<br>8.7% other or multiple race/ethnicity<br><br>33.3%: Ever use of e-cigarettes	• Experiences with e-cigarettes<br>• Comparison between e-cigarettes and cigarettes<br>• Social norms around e-cigarette use<br>Recordings supplemented by field notes		
**4**	Case et al (7) 2016 Southwestern University, Texas, USA	N=30<br>Age: 18-26<br>Females:16<br>Males:14<br><br>40% white non-Hispanic<br>17% Hispanic<br>30% Asian,<br>13% other races<br><br>50%: Current e-cigarette users	Purposive sampling of e-cigarette users and non -users from university participant pool and recruitment online through university wide event calendar<br>Semi structured interviews<br>Interview guide developed from the 'Health Belief Model' (8,9,10) and the 'Theory of Planned Behaviour' (11,12)<br>Topic guide centred around: knowledge, attitudes, perceived benefits/advantages, perceived barriers, perceived disadvantages, perceived norms and perceived self-efficacy	Thematic analysis (13)	Good
**5**	Chen et al (14)<br>2019<br>Washing D.C., Maryland and Virginia, USA	N=25<br>Age 18-34<br><br>Female: 10<br>Male:15<br><br>36% non-Hispanic black<br>28% non-Hispanic white<br>20% mixed race<br>8% white<br>8% other races	Participants recruited from posting on Craigslist.com<br>Semi-structured interviews conducted over the phone<br>No mention how topic guide was developed<br>Topic guide centred around:<br>• Young adult smokers' attitudes and beliefs towards e-cigarette flavours<br>• Intentions of using e-cigarettes given an e-cigarette flavour ban	Thematic analysis (15)	Good

		100%: Used e-cigarettes in the past 30 days			
**6**	Cheney et al (16)<br>2016<br>Oklahoma, USA	N= 30<br>Age: 18-30<br>Females:16<br>Male:14<br><br>77% white<br>17% African American<br>3% Hispanic<br>3% American Indian<br><br>77%: daily e-cigarette use<br>15%: use e-cigarette at least once a week<br>10%: use e-cigarette at least once a month	Purposive sampling of e-cigarette users through posters within the local community and online advertising<br>Semi-structured interviews<br>Interview questions developed from a review of the literature, observations in vape stores and conversations with three straight-to-work young adults who were not included in the study<br>Questions included were:<br>• Tell me about the first time you used an e-cigarette<br>• How much did you vape when you started<br>• How much do you use now<br>• If you run out of juice and the stores are closed, what do you do<br>• How do your family and friends view vaping<br>• Do people view you differently when they see you vaping rather than smoking<br>• Looking back, how do you feel about your decision to start vaping<br>• Do you see yourself quitting vaping at some point	Thematic analysis (17)	Good
**7**	Cheney et al (18)<br>2018<br>Southwest USA	N= 32<br>Age:18-25<br><br>Female: 10<br>Male: 22<br><br>75% white<br>3% African-American<br>13% Hispanic<br>3% Asian<br>6% American Indian	Purposive sampling of participants through recruitment posters placed on university campuses and postings on website visited by students<br>Semi-structured interviews<br>Topic guide developed through reviewing literature and prior interviews with a small sample of college student e-cigarette users who were not included in the study<br>Topic guide centred around:<br>• Prior and current use of e-cigarettes and other tobacco products<br>• How and when e-cigarettes are used<br>• Beliefs about e-cigarettes<br>• Specific influences on e-cigarette use<br>• How family members and peers viewed their e-cigarette use	Thematic analysis (19)	Good

		100%: use e-cigarette once a week			
**8**	Choi et al (20)<br>2012<br>Minneapolis, Minnesota, USA	N=66<br>Age: 18-26<br>Female:40<br>Male: 26<br><br>56% American White<br>29% Asian<br>6% African American<br>1% Other	Recruited through online adverts, flyers at two college campuses, a recruitment booth and an announcement in a student newsletter<br>11 focus groups stratified to gender and education<br>Discussion guide developed through a literature review and with experts in tobacco control policy, marketing, advertising and social media<br>Topic guide centred around: Perceptions of new tobacco products and experiences of these products.<br>Participants were also shown pictures of snus, dissolvable tobacco products and e-cigarettes	Thematic analysis (21)	Good
**9**	Coleman et al (22)<br>2016<br>Washington, Orlando, Providence, Richmond and Los Angeles. USA	N=116<br>Age: 18-29 + 30><br>Female:57<br>Male:59<br><br>66% American White<br>15% Black or African American<br>11% Hispanic<br>7% Asian<br>1% American Indian<br>2% Other<br><br>47%: exclusively used e-cigarettes in the past 30 days<br>53%: used e-cigarettes as well as one other	Recruitment of participants through local market research firms<br>14 Focus groups<br>Moderator guide developed from the 'Theory of Planned Behaviour' (23)<br>Questions included:<br>• Why they used e-cigarette products<br>• The benefits of using them<br>• Similarities and differences between e-cigarettes and other tobacco products<br>• Knowledge about ingredients<br>• Beliefs about the harm of e-cigarettes<br>• Their friends and families' opinions of e-cigarettes	A phenomenological approach (24)	Good

		tobacco product in the past 30 days			
**10**	Cooper et al (25)<br>2016<br>University of Texas Health Science Centre. Austin, Texas, USA	N= 59<br>Age: 21-23 + 37-41<br>Female:26<br>Male:24<br><br>56% American White<br>6% African American<br>22% Asian<br>16% Other<br><br>72%: use e-cigarettes daily<br>28%: use e-cigarettes some days	Recruitment through a research opportunity post on the university online calendar<br>Interview guide developed through review of existing literature<br>Semi-structured interviews<br>Interview guide centred around:<br>• Motivations for use and perceptions of health impact<br>• Subjective effects of nicotine delivery and dependence<br>• Self-reported efficacy for smoking cessation or reduction	Thematic analysis (26)	Good
**11**	De Andrade et al (27)<br>2016<br>Fife, Scotland	N=182<br>Ages 13-16	Recruited purposively by sex and smoking status<br>Health education and promotion programme produced a 'Pop-Up Radio Project' to explore whether pupils recalled key points from tobacco-education interventions<br>E-cigarettes were discussed by pupils<br>All pupils were interviewed by the primary investigator<br>Questions centred around:<br>• Whether they had taken part in any interventions<br>• Gathering perceptions of interventions if applicable<br>• Perceptions of smoking<br>• Assessing views on smoking prevalence in young people<br>• Exploring how interventions can be improved	Thematic analysis (26)	Good
**12**	Dobbs et al (28)<br>2019<br>Arkansas USA	N=22<br>Age: 18+<br><br>Female:11<br>Male:11	Participants recruited through campus online newsletter<br>5 Focus groups<br>Topic guide developed from reviewing existing literature.<br>Topic guide centred around:	Thematic analysis (26)	Good

		Demographic data not collected from participants<br><br>45%: trying/using e-cigarettes	• The acceptability of college students using traditional cigarettes and e-cigarettes<br>• Where individuals typically used e-cigarettes<br>• Exploring social norms for cigarette and e-cigarette<br>• Assessing participants knowledge and opinions of tobacco-free policies		
**13**	Dono et al (29)<br>2019<br>South Australia	N=30<br>Age:18-25<br><br>Females:13<br>Male:17<br><br>33%: Never used e-cigarettes<br>47%: Experimental use of e-cigarettes<br>20%: Used e-cigarettes to quit smoking	Participants recruited using a professional recruitment company<br>6 Focus groups<br>No mention how topic guide was developed<br>Visual stimuli used to facilitate discussions<br>Topic guide centred around:<br>• Experiences with smoking tobacco<br>• Thoughts on smoking cessation<br>• Knowledge and perceptions of e-cigarettes<br>• Perceptions of messages portrayed in e-cigarette advertising<br>• Perceptions of anti-e-cigarette messaging in the media	Thematic analysis (30)	Good
**14**	Gowin et al (31)<br>2017<br>Oklahoma, USA	N=30<br>Age:19-31<br><br>Females:14<br>Males:14<br><br>77%: White<br>17%: African American<br>3%: Hispanic<br>3%: American Indian<br><br>100%: Current users of e-cigarettes	Purposive sample of participants recruited through posters at various community locations<br>Semi structured interviews<br>Topic guided developed from review of current literature, author's research on e-cigarette store owners and conversations with a small sample of young adults who were not included in the study<br>Topic guide centred around:<br>• Knowledge, beliefs and rules regarding the health/safety of e-cigarette<br>• Where they accessed their information	Grounded theory (32)	Good

**15**	Hammal and Finegan (33)<br>2016<br>University of Alberta, Canada	N= 45<br>Age: 12-17<br>Female:22<br>Male:23<br><br>33% European<br>13.3% Asian<br>4.4% Latin American<br>35.6% African American<br>13.3% Other<br><br>0%: used e-cigarettes in the past 30 days<br>54.9%: ever use of e-cigarettes	Convenience and quota sampling within schools to recruit participants along with snowball sampling<br>8 Focus groups with participants stratified by age<br>Topic guide developed from literature review<br>Questions centred around knowledge, initiation, use behaviour and family attitudes.	Content analysis (32)	Good
**16**	Hardcastle et al (34)<br>2014<br>Chester and Merseyside. England, UK	N=45<br>Age:13-17<br>Female: 15<br>Male: 30<br><br>91.1%: White British<br>8.99%: Other<br><br>42.2%: ever use of e-cigarettes	Recruitment was facilitated by youth group leaders and teachers<br>7 Focus groups<br>Development of topic guide not discussed<br>Topic guide centred around young people's use of and attitudes towards e-cigarettes	Thematic analysis (26)	Good
**17**	Harrell et al (35)<br>2019<br>Southeastern USA	N= 49<br>Age: 18-29<br><br>Female:15<br>Male:34	Participants recruited through publicised adverts<br>4 focus groups and 2 individual interviews<br>Topic guide developed from reviewing current literature<br>Topic guide centred around:<br>• E-cigarette expectancies among young adults<br>• Exploring beliefs regarding social effects of e-cigarettes<br>• Perceptions about short/long term effects of e-cigarettes	Content analysis (36)	Good

		44.9% Non-Hispanic white<br>30.6% Non-Hispanic African American<br>8.2% Non-Hispanic Asian<br>4.1% Non-Hispanic multiracial<br>12.2% Hispanic<br><br>Nonuser: 44.9%<br>E-cigarette user: 24.5%<br>Dual User: 16.3%<br>Cigarette user: 14.3%			
**18**	Hess et al (37)<br>2017<br>San Francisco Bay area, California, USA	N=46<br>Age: 18-25<br>Female:24<br>Male:22<br><br>100%: African American<br><br>33%: ever use of e-cigarettes	Recruited through social media and in local communities<br>Further snowball sampling used<br>Structured interviews<br>The interview guide was developed from a literature search and exploratory focus groups conducted with black young adults<br>Interview questions centred around:<br>• Background of the participant<br>• Racial identity<br>• Personal tobacco and e-cigarette use<br>• Smoking identity<br>• Beliefs about tobacco<br>• The social acceptability of tobacco<br>• Perceptions and use of e-cigarettes<br>Recordings supplemented by field notes	Thematic analysis (26)	Good
**19**	Hilton et al (38)<br>2016<br>Scotland and	N=83<br>Age: 14-17<br>Female:39<br>Male:44	Recruited purposively by SES, gender, conventional cigarette use and e-cigarette use<br>16 focus groups<br>Topic guide developed from a literature search and pilot work	Thematic analysis (26)	Good

	England.<br>UK	45%: ever use of e-cigarettes<br>11%: Current e-cigarette users	The guide focused on 5 areas, which were knowledge and understanding about e-cigarettes, belief about the potential benefits and harms, experiences of e-cigarettes, knowledge and opinions on regulation<br>Discussions further facilitated by images of e-cigarettes and promotional material<br>Recordings supplemented by field notes		
**20**	Johnson et al (39)<br>2017<br>Georgetown University Medical Centre, Washington, DC, USA	N=25<br>Age: 12-17<br>Female:14<br>Male:11<br><br>32%: African American<br>52%: White<br>16%: Other<br><br>8%: ever use of e-cigarettes	Purposive sampling of participants from an adolescent medicine clinic in an urban tertiary care hospital<br>Semi-structured interviews<br>Topic guide was developed from a literature review and consultation with study team<br>Questions centred around:<br>• Exposure to e-cigarettes<br>• Recall of details of e-cigarette advertising locations<br>• Perceived harm and addictiveness of e-cigarettes<br>• Features of e-cigarettes and their advertising that may enhance or detract from their appeal to youths	Thematic analysis (26)	Good
**21**	Keamy-Minor et al (40)<br>2019<br>California, USA	N= 24<br>Age: 18-29<br><br>Female: 5<br>Male:19<br><br>Demographic data not recorded<br><br>100%: Current e-cigarette user	Participants recruited through posts on Facebook and Instagram<br>Semi-structured interview<br>No mention how topic guide was developed<br>Topic guide centred around:<br>• How young adults perceive and use next generation pod device<br>• If their use of pod devices differs from non-pod e-cigarettes and traditional cigarettes	Thematic analysis (41)	Good
**22**	Lucherini et al (42)<br>2018<br>Scotand, UK	N= 72<br>Age:16-24<br><br>Female: 39<br>Male: 33<br><br>Demographic data not recorded	Participants recruited through community organisation, educational institutions, postings on skills exchange website Gumtree and snowball methods.<br>22 focus groups and 11 individual semi-structured interviews<br>No mention how topic guide was developed<br>Topic guide centred around:<br>• Smoking/vaping history<br>• Where, when and how participants had encountered e-cigarettes	Thematic analysis (26)	Good

		83%: Ever use of e-cigarettes	Different e-cigarette types were brought as props to generate discussions<br>Visual example of e-cigarettes being used and e-cigarette advertising were provided during the interview		
**23**	Lucherini et al (43)<br>2019<br>Scotland, UK	N= 72<br>Age:16-24<br><br>Female: 39<br>Male: 33<br><br>Demographic data not recorded<br><br>83%: Ever use of e-cigarettes]	Purposive sample of participants recruited through community organisation, educational institutions, postings on skills exchange website Gumtree and snowball methods.<br>22 focus groups and 11 individual semi-structured interviews<br>No mention how topic guide was developed<br>Topic guide centred around:<br>• Experiences of smoking and vaping<br>• Participants experiences of e-cigarette marketing and advertising<br>Different e-cigarette types were brought as props to generate discussions<br>Visual example of e-cigarettes being used and e-cigarette advertising were provided during the interview	Thematic analysis (26)	Good
**24**	Maglalang et al (44)<br>2019<br>California, USA	N= 12<br>Age: 19-34<br><br>Female: 4<br>Male: 8<br><br>100%: Asian American<br><br>100%: Current e-cigarette users	Participants recruited through social media, electronic newsletters and snowball sampling<br>Semi-structured interviews<br>Topic guide developed from review of existing literature<br>Topic guide centred around:<br>• Their identity<br>• Use of Electronic cigarettes<br>• Health perceptions of electronic cigarettes<br>How Asian culture influences behaviour and attitudes towards Electronic Cigarettes	Thematic analysis (26)	Good
**25**	McDonald EA and Ling PM (45)<br>2015<br>Manhattan, Brooklyn and Queens. NYC, USA	N=87<br>Age: 18-27<br>Female:44<br>Male:56<br><br>52%: Non-Hispanic white<br>9%: African American	Recruitment of participants from bars<br>12 Semi-structured interviews<br>16 Focus group stratified to either daily smokers or non-smokers who had used e-cigarettes<br>No mention on how topic guide was developed<br>Topic guide centred around:<br>• Definitions of smoking and smokers<br>• Experiences with tobacco and e-cigarettes<br>• Perceived benefits and risks<br>• Experiences with stopping tobacco use	Thematic analysis (26)	Good

		3%: Pacific Islander<br>23%: Hispanic<br>13%: Other races<br><br>32%: current e-cigarettes users	Recording supplemented with video-taping of focus groups		
**26**	McKeganey et al (46)<br>2018<br>North of England, Scotland, UK	N=8<br>Age: 16-30<br><br>Female:2<br>Male: 6<br><br>Demographic data not recorded	Participants recruited from larger e-cigarette study<br>Two semi-structured interviews occurred 6 months apart<br>No mention how topic guide was developed<br>Topic guide centred around:<br>• Perceptions and use of e-cigarette<br>• Circumstances of their use<br>• How their use is perceived by others<br>• The frequency of their use of e-cigarettes<br>• The likelihood of participants using e-cigarettes in the future	Content analysis<br>No reference included	Fair
**27**	McKeganey et al (47)<br>2018<br>North of England, Scotland, UK	N=50<br>Age:16-26<br><br>Females:18<br>Male:32<br><br>100%: E-cigarette users	Participants recruited from universities/colleges, leisure and work settings and snowball sampling.<br>No mention how topic guide was developed<br>Topic guide centred around:<br>• Reasons for initiating e-cigarette use<br>• Views on the relative harm of smoking and vaping<br>• Views on how similar or dissimilar the activities of vaping and smoking are<br>• Whether in their view their likelihood of smoking had increased or decreased as a result of their vaping	Content analysis<br>No reference included	Good
**28**	Peters et al (48)<br>2013<br>University of Houston, Texas, USA	N= 47<br>Age: 15-17<br>Male: 47<br><br>74%: African American<br>26%: Hispanic American	Purposive sampling of male participants by school staff<br>No mention on how topic guide was developed<br>Focus groups stratified by age<br>Questions asked:<br>• Why do youth use electronic cigarettes<br>• Where are places that you use electronic cigarettes<br>• What do your friends think about electronic cigarettes<br>• Why are electronic cigarettes so popular	Thematic analysis (26)	Fair

		100%: current e-cigarette users	Recordings supplemented by field notes		
**29**	Pokhrel et al (49)<br>2015<br>University of Hawaii Cancer Centre, Hawaii, USA	N=62<br>Age: 18-35<br>Female:39<br>Male:23<br><br>19%: Asian American<br>8%: African American<br>12%: Filipino<br>7%: Hispanic/Latino<br>23%: Native Hawaiian/other Pacific Islander<br>31%: White<br><br>100%: current e-cigarette users	Recruitment through adverts and flyers at colleges<br>12 mix-gender focus groups<br>Topic guide development not mentioned<br>Three sets of questions asked across all focus groups<br>The responses to one set of questions is discussed in this paper<br>• If you currently smoke cigarettes when do you smoke cigarettes and when do you use e-cigarette<br>• [For those who had quit both] At the time when you were using both cigarettes and e-cigarettes, when did you use cigarettes and when did you use e-cigarettes<br>Recording supplemented by field notes	Content analysis (50)	Good
**30**	Pokhrel et al (51)<br>2015<br>University of Hawaii Cancer Centre, Hawaii, USA	N= 62<br>Age: 18-35<br>Female:23<br>Male:39<br><br>19%: Asian American<br>8%: African American<br>12%: Filipino<br>7%: Hispanic/Latino<br>23%: Native Hawaiian/other Pacific Islander<br>31%: White	Recruitment through adverts and flyers at colleges<br>12 mix-gender focus groups<br>Topic guide development not mentioned<br>Three sets of questions were asked across the focus groups<br>The responses to two sets are discussed in this paper<br>• What do you like about e-cigarettes<br>• Why do you use e-cigarettes<br>• What are the positive consequences that you expect out of using e-cigarettes<br>• What do you not like about e-cigarettes<br>• What are the negative consequences that you expect out of using e-cigarette<br>• What are the reasons that may make you stop using e-cigarettes<br>• Questions on dual use were asked but not provided as it is not relevant to this study<br>Recording supplemented by field notes	Content analysis (50)	Good

		100%: current e-cigarette users			
**31**	Roditis et al (52)<br>2015<br>Stanford University, California, USA	N=24<br>Age: 15-19<br>Female: 9<br>Male:15<br><br>25%: White<br>33.3% Black/African American<br>16.7%: Asian<br>4.2%: American Indian/Alaskan native<br><br>16.7%: ever use of e-cigarettes	Participants were purposefully selected from afterschool programmes in urban Northern California school district<br>6 Focus groups<br>Topic guide development not mentioned<br>Semi-structured guide used to assess perceptions of the risk and benefits related to using conventional cigarettes, e-cigarettes and marijuana:<br>• Where, what and from whom they had learned about these products<br>• Asked to discuss why they would use one product over another product	Thematic analysis (26)	Good
**32**	Wagoner et al (53)<br>2016<br>Raleigh, Durham and Chapel Hill. North Carolina, USA	N=77<br>Age: 13-25<br>Female:43<br>Male:34<br><br>57%: White<br>26%: Black<br>4%: Asian<br>13%: Other<br><br>28.6%: past 30-day e-cigarette use<br>14.3%: ever use of e-cigarettes	Recruitment through adverts on social media, newspapers, and media outlets<br>Further in-person recruitment in community settings<br>10 Focus groups stratified by age and use of e-cigarettes<br>Topic guide developed from a literature review, pilot work and input from the study team<br>Topic guide centred around familiarity, knowledge and health effects<br>New questions added to focus groups 7-10<br>• Have you heard of e-hookahs or hookah pens<br>• What do you know about them<br>• Are they different from e-cigarettes<br>• Do you know people who use them<br>• Are there other devices like this we should know about	Thematic analysis (26)	Good
**33**	Weishar et al (54)<br>2016	N=83<br>Age: 14-17<br>Female:39<br>Male:44	Recruited purposively by age, SES, smoking status and e-cigarette use<br>16 Focus groups<br>Topic guide developed from a literature search and pilot work	Thematic analysis (26)	Good

	Scotland and England. UK	57%: ever use of e-cigarettes	The guide focused on five areas, which were, knowledge and understanding about e-cigarettes; belief about the potential benefits and harms; experiences of e-cigarettes; knowledge and opinions on regulation<br>Discussions further facilitated by images of e-cigarettes and promotional material<br>Recordings supplemented by field notes		
**34**	Yule and Tinson (55) 2017 Massachusetts, USA	N=20<br>Age: 21-27<br>Female:12<br>Male:8<br><br>45%: current e-cigarette users	Purposive sampling of participants in 'emerging adulthood' who engage with vaping behaviour<br>Semi-structured interviews<br>Topic guide/question development process not mentioned<br>Questions centred around:<br>• Experiences and perspectives of e-cigarettes<br>• Sociability of vaping<br>• Knowledge of vaping<br>• Thoughts on restrictions and regulations on e-cigarettes	Grounded theory (56)	Good

1. Akre C, Suris JC. Adolescents and young adults' perceptions of electronic cigarettes as a gateway to smoking: a qualitative study in Switzerland. Health Educ Res. 2017;32(5):448–54. https://doi.org/10.1093/her/cyx054
2. Collingridge DS, Gantt EE. The quality of qualitative research. Am J Med Qual. 2008;23(5):389–95. https://doi.org/10.1177/1062860608320646.
3. Antin T, Hess C, Kaner E, Lipperman-Kreda S, Annechino R, Hunt G. Pathways of nicotine product use: a qualitative study of youth and young adults in California. Nicotine Tob Res. 2020;22(5):722–7. https://doi.org/10.1 093/ntr/ntz028.
4. Bernard HR. Analyzing qualitative data: systematic approaches. Los Angeles; London: SAGE; 2010.
5. Camenga DR, Cavallo DA, Kong G, Morean ME, Connell CM, Simon P, et al. Adolescents' and young adults' perceptions of electronic cigarettes for smoking cessation: a focus group study. Nicotine Tob Res. 2015;17(10): 1235–41. https://doi.org/10.1093/ntr/ntv020.
6. Ritchie J, Lewis J. Qualitative research practice: a guide for social science students and researchers. London: SAGE Publications; 2003.
7. Case K, Crook B, Lazard A, Mackert M. Formative research to identify perceptions of e-cigarettes in college students: implications for future health communication campaigns. J Am Coll Heal. 2016;64(5):380–9. https:// doi.org/10.1080/07448481.2016.1158180.
8. Rosenstock IM. What research in motivation suggests for public health. Am J Public Health Nations Health. 1960;50(3_Pt_1):295–302. https://doi.org/1 0.2105/AJPH.50.3_Pt_1.295
9. Rosenstock IM. The health belief model and preventive health behavior. Health Educ Behav. 1974;2(4):354–86. https://doi.org/10.1177/1090198174 00200405.

10. Champion VL, Skinner CS. The health belief model. 4th ed. San Francisco: Jossey-Bass; 2008
11. Ajzen I, M F. Understanding attitudes and predicting social behaviour. N. J, USA: Prentice-Hall; 1980
12. Fishbein MA, Ajzen I. Belief, attitude, intention and behaviour: an introduction to theory and research. MA, USA: Addison-Wesley; 1975
13. Miles MB, Huberman MA, Saldana J. Qualitative data analysis: a methods sourcebook. 3rd ed. Thousand Oaks: SAGE Publications; 2013.
14. Chen JLC, Green K, Fryer C, Borzekowski D. Perceptions about e-cigarette flavors: a qualitative investigation of young adult cigarette smokers who use e-cigarettes. Addict Res Theory. 2019;27(5):420–8. https://doi.org/10.1080/1 6066359.2018.1540693.
15. Rice PL. Qualitative research methods: a health focus. Victoria: Oxford University Press; 1999.
16. Cheney MK, Gowin M, Wann TF. Electronic cigarette use in straight-to-work young adults. Am J Health Behav. 2016;40(2):268–79. https://doi.org/10. 5993/AJHB.40.2.12
17. Miles MB, Huberman MA, Saldana J. Qualitative data analysis: a methods sourcebook. 3rd ed. Thousand Oaks: SAGE Publications; 2013
18. Cheney MK, Gowin M, Clawson AH. Using the ecological model to understand influences on college student vaping. J Am Coll Heal. 2018; 66(7):597–607. https://doi.org/10.1080/07448481.2018.1440578.
19. Tolley EE, Ulin PR, Mack N, Robinson ET, Succop SM. Qualitative methods in public health: a field guide for applied research. 2nd ed. Hoboken: Wiley; 2016.
20. Choi K, Fabian L, Mottey N, Corbett A, Forster J. Young adults' favorable perceptions of snus, dissolvable tobacco products, and electronic cigarettes: findings from a focus group study. Am J Public Health. 2012;102(11):2088– 93. https://doi.org/10.2105/AJPH.2011.300525
21. Boyatzis RE. Transforming qualitative information: thematic analysis and code development. Thousand Oaks: SAGE Publications; 1998.
22. Coleman BN, Johnson SE, Tessman GK, Tworek C, Alexander J, Dickinson DM, et al. "It's not smoke. It's not tar. It's not 4000 chemicals. Case closed": Exploring attitudes, beliefs, and perceived social norms of e-cigarette use among adult users. Drug Alcohol Depend. 2016;159:80–5.
23. Icek A. The theory of planned behaviour. Organ Behav Hum Decis Process. 1991;50(2):179–221. https://doi.org/10.1016/0749-5978(91)90020-T.
24. Giorgi A. The theory, practice, and evaluation of the phenomenological method as a qualitative research procedure. J Phenomenol Psychol. 1997; 28(2):235–60. https://doi.org/10.1163/156916297X00103.
25. Cooper M, Harrell MB, Perry CL. Comparing young adults to older adults in e-cigarette perceptions and motivations for use: implications for health communication. Health Educ Res. 2016;31(4):429–38. https://doi.org/10.1 093/her/cyw030.
26. Braun V, Clarke V. Using thematic analysis in psychology. Qual Res Psychol. 2006;3(2):77–101. https://doi.org/10.1191/1478088706qp063oa

27. de Andrade M, Angus K, Hastings G. Teenage perceptions of electronic cigarettes in Scottish tobacco-education school interventions: co-production and innovative engagement through a pop-up radio project. Perspect Public Health. 2016;136(5):288–93. https://doi.org/10.1177/1757913915612109.
28. Dobbs PD, Rolfe DT, Cheney MK. Cigarettes vs. E-cigarettes: policy implications from a focus group study. Subst Use Misuse. 2019;54(12):2082– 7. https://doi.org/10.1080/10826084.2019.1626436
29. Dono J, Wilson C, Ettridge K, Miller C. 'I don't think I'd feel good about myself if I was to give up smoking and go to one of these': perceptions of e-cigarettes among south Australian young adult smokers and ex-smokers. Addict Res Theory. 2019;27(3):258–68. https://doi.org/10.1080/16066359.201 8.1501032.
30. Braun V, Clarke V. Successful qualitative research : a practical guide for beginners. London: Los Angeles: SAGE; 2013
31. Gowin M, Cheney MK, Wann TF. Knowledge and beliefs about e-cigarettes in straight-to-work young adults. Nicotine Tob Res. 2017;19(2):208–14. https://doi.org/10.1093/ntr/ntw195
32. Patton MQ. Qualitative research and evaluation methods. 3rd ed. Thousand Oaks: Sage Publications; 2002.
33. Hammal F, Finegan B. Exploring attitudes of children 12-17 years of age toward electronic cigarettes. J Community Health. 2016;41(5):962–8. https:// doi.org/10.1007/s10900-016-0178-6.
34. Hardcastle K, Hughes K, Worsley J, Bennett A, Ireland R, Sweeney S. "Most people I know have got one" - young people's perceptions and experiences of electronic cigarettes summary report. Liverpool: Health Equalities Group; 2014
35. Harrell PT, Brandon TH, England KJ, Barnett TE, Brockenberry LO, Simmons VN, et al. Vaping expectancies: a qualitative study among young adult nonusers, smokers, vapers, and dual users. Subst Abuse Res Treat. 2019;13: 1178221819866210.
36. Bridges S, Jin J. Qualitative research in PBL in health sciences education: a review. Interdiscip J Probl Based Learn. 2016;10(2). https://doi.org/10.7771/1 541-5015.1605
37. Hess CA, Antin TMJ, Annechino R, Hunt G. Perceptions of E-cigarettes among black youth in California. Int J Environ Res Public Health. 2017;14(1): 60. https://doi.org/10.3390/ijerph14010060.
38. Hilton S, Weishaar H, Sweeting H, Trevisan F, Katikireddi SV. E-cigarettes, a safer alternative for teenagers? A UK focus group study of teenagers' views. BMJ Open. 2016;6(11):e013271. https://doi.org/10.1136/bmjopen-2016-0132 71.
39. Johnson AC, Mays D, Hawkins KB, Denzel M, Tercyak KP. A qualitative study of adolescent perceptions of electronic cigarettes and their marketing: implications for prevention and policy. Childrens Health Care. 2017;46(4): 379–92. https://doi.org/10.1080/02739615.2016.1227937.
40. Keamy-Minor E, McQuoid J, Ling PM. Young adult perceptions of JUUL and other pod electronic cigarette devices in California: a qualitative study. BMJ Open. 2019;9(4):e026306. https://doi.org/10.1136/bmjopen-2018-026306
41. McQuoid J, Keamy-Minor E, Ling PM. A practice theory approach to understanding poly-tobacco use in the United States. Crit Public Health. 2020;30(2):204–19. https://doi.org/10.1080/09581596.2018.1541226
42. Lucherini M, Rooke C, Amos A. E-cigarettes, vaping and performativity in the context of tobacco denormalisation. Sociol Health Illn. 2018;40(6):1037– 52. https://doi.org/10.1111/1467-9566.12741.

43. Lucherini M, Rooke C, Amos A. "They're thinking, well it's not as bad, I probably won't get addicted to that. But it's still got the nicotine in it, so...": maturity, control, and socializing: negotiating identities in relation to smoking and vaping - a qualitative study of young adults in Scotland. Nicotine Tob Res. 2019;21(1):81–7. https://doi.org/10.1093/ntr/ntx245
44. Maglalang DD, Le MN, Yoo GJ, Del Mundo GO. Personal motivations of Asian Americans who use ENDS: a qualitative study. Am J Health Behav. 2019;43(4):680–90. https://doi.org/10.5993/AJHB.43.4.3
45. McDonald EA, Ling PM. One of several 'toys' for smoking: young adult experiences with electronic cigarettes in New York City. Tob Control. 2015; 24(6):588–93. https://doi.org/10.1136/tobaccocontrol-2014-051743.
46. McKeganey N, Barnard M. Change and continuity in vaping and smoking by young people: a qualitative case study of a friendship group. Int J Environ Res Public Health. 2018;15(5):1008. https://doi.org/10.3390/ijerph15051008.
47. McKeganey N, Barnard M, Russell C. Vapers and vaping: E-cigarettes users views of vaping and smoking. Drugs. Educ Prev Policy. 2018;25(1):13–20. https://doi.org/10.1080/09687637.2017.1296933.
48. Peters RJ Jr, Meshack A, Lin MT, Hill M, Abughosh S. The social norms and beliefs of teenage male electronic cigarette use. J Ethn Subst Abus. 2013; 12(4):300–7. https://doi.org/10.1080/15332640.2013.819310.
49. Pokhrel P, Herzog TA, Muranaka N, Regmi S, Fagan P. Contexts of cigarette and e-cigarette use among dual users: a qualitative study. BMC Public Health. 2015;15(1):859. https://doi.org/10.1186/s12889-015-2198-z
50. Elo S, Kyngas H. The qualitative content analysis process. J Adv Nurs. 2008; 62(1):107–15. https://doi.org/10.1111/j.1365-2648.2007.04569.x.
51. Pokhrel P, Herzog TA, Muranaka N, Fagan P. Young adult e-cigarette users' reasons for liking and not liking e-cigarettes: a qualitative study. Psychol Health. 2015;30(12):1450–69. https://doi.org/10.1080/08870446.2 015.1061129.
52. Roditis ML, Halpern-Felsher B. Adolescents' perceptions of risks and benefits of conventional cigarettes, E-cigarettes, and marijuana: a qualitative analysis. J Adolesc Health. 2015;57(2):179–85. https://doi.org/10.1016/j.jadohealth.201 5.04.002.
53. Wagoner KG, Cornacchione J, Wiseman KD, Teal R, Moracco KE, Sutfin EL. Ecigarettes, hookah pens and vapes: adolescent and young adult perceptions of electronic nicotine delivery systems. Nicotine Tob Res. 2016; 18(10):2006–12. https://doi.org/10.1093/ntr/ntw095
54. Weishaar H, Trevisan F, Hilton S. Maybe they should regulate them quite strictly until they know the true dangers': a focus group study exploring UK adolescents' views on e-cigarette regulation. Addiction. 2016;111(9):1637–45. https://doi.org/10.1111/add.13377.
55. Yule JA, Tinson JS. Youth and the sociability of "vaping". J Consum Behav. 2017;16(1):3–14. https://doi.org/10.1002/cb.1597
56. Juliet C, Anselm S. Basics of qualitative research:grounded theory. Newbury Park: SAGE Publishing House; 1990.
