# Supplementary material for: “That’s the whole thing about vaping, it’s custom tasty goodness”: a meta-ethnography of young adults’ perceptions and experiences of e-cigarette use
Source: Subst Abuse Treat Prev Policy. 2021 Nov 12;16:85. doi: 10.1186/s13011-021-00416-4 (PMC8586839; doi:10.1186/s13011-021-00416-4)
Supplement: Supplementary file 3 — Additional file 3: Table S1. Summary of 3rd order constructs and participant quotes. [file 13011_2021_416_MOESM3_ESM.pdf]

Table 1 Summary of 3rd order constructs and participant quotes

Socio-ecological model	Meta theme (3rd order construct)	1st order data (participant quotes)
**1. Individual use and identity**	1.1 E-cigarettes promote positive self-identity	• *"Oh, e-cigarettes are classy, because you can walk around with them. They do not have any vapor that goes around and they look nice."*- Demographic not recorded (34)
	1.2 Expressing individual identity through e-cigarette choice	• *"Flavours – if I don't want a menthol or tobacco taste but I still want a bit of nicotine I can go dragon berry or peanut butter gumdrop chew or one of my favourites, which I like is my own mix it's pretty good it's blueberry and marshmallow… that is the whole thing about vaping it's custom tasty goodness."*- Demographic not recorded (27)
	1.3 E-cigarettes as a playful hobby	• *"It's almost like being a kid with a toy. And the way you have to put the oils in some of them, you have to break it down, and take it, and then take the juice and put the little thing and concentrate on it."* - Male, 25 years old (35)<br>• *"I have phases where I'll go through a lot of the e-juice at the one time and then I'll just go off it for a wee bit and then just have the occasional one at night…. When I was studying over the prelims, I went through so many bottles because I hate studying and it was just something to do.*" - Male, 16 years old, traditional cigarette user, e-cigarette user (50)
	1.4 E-cigarettes a safer alternative to smoking	• "[…….] *And I know a friend who started on 10, and it's a goal for them, it's like weight-watchers or something. He comes up to me like, "Hey bro! I made it down to 8!" He's so proud of himself, and now he's on 4 so … it's self-rewarding, psychologically."* - Male, 25 years old (35)<br>• *"I tried to stop smoking with a vapouriser but it only went so far… It doesn't work when you out drinking."* - Male, 19 years old, traditional cigarette user, ex-e-cigarette user (42)<br>• *"I felt a lot more energetic,* [using e-cigarettes] *a lot more healthy, less out of breath like I can go running again you know versus what I did when I was smoking I mean I couldn't do it cause I couldn't breathe."*- Female, 25 years old, ex- traditional cigarette user (28)
	1.5 Excessive use and addiction to e-cigarettes	• *"I know people who, you know, like, will hit their vape once. And I know people who don't breathe oxygen right? Like, they only use their vape."*- Male, 23 years old, tried e-cigarettes, traditional cigarette user (43)<br>• *"You kinda reach a natural end of…'right, I've been using this for four minutes, that's like a fag, I'm going to put this away now,' so it was weird to see someone just sitting constantly* [vaping]. *"* - Female, 22 years old, traditional cigarette user, tried e-cigarettes (42)
	1.6 E-cigarettes a risk factor for subsequent tobacco use	• *"The electronic cigarette can make the gesture a commonplace, one will lose track of the danger of smoking by starting with the [electronic cigarette] just for the taste* […] *and after why not pass on to [traditional cigarettes] which is the following step."* - Male, 19 years old, traditional cigarette user (61)
**2.Social use and identity**	2.1 E-cigarettes provide social status and group acceptance	• *"……. breaking the ice in terms of conversation, you have something that you all have in common. You can talk about your different flavours, the brand. There is a history and a commonality between other people*." - Male (36)
	2.2 Relative acceptability or stigma of e-cigarettes compared to traditional cigarettes	• *"…when you are sitting next to a person that doesn't smoke like at the bus stop and you are vaping, they don't get up and move. When you are smoking they get up and move."*- Demographic not recorded (27)
	2.3 Context-dependent e-cigarette use	• *"… being on 24 hour on call last thing you want to do is show up smelling like an ash tray."*-Demographic not recorded (27)
	2.4 Negative stereotypes of e-cigarette users	• *"People just kind of mess with you a bit when you've got it, people can undermine you a bit and say, "Ha, what a gimp he's vaping, you know."* - Male, 16 years old, e-cigarette user, traditional cigarette user (50)<br>• *"So I went and bought an e-cigarette [larger device], and the I felt really awkward using e-cigarettes cause they're douche, and so I got a JUUL because JUULs for some reason aren't douchey."*- Male, 21 years old, e-cigarette user (43)<br>• *"I'm not addicted to cigarettes. I can smoke for, say, like a year, like consistently, every day have a fag… I don't get addicted"* - Female, 17 years old, traditional cigarette user, tried e-cigarettes (42)
	2.5 School and family contexts supporting e-cigarette use	• *"My mother* [would approve of e-cigarette use]. *She would rather me use an e-cigarette than conventional cigarettes, that's for sure. People that care about*

		me would rather me smoke an e-cig than smoke conventional cigarettes." - Female, e-cigarette user (29)
**3.E-cigarette marketing and availability**	3.1 E-cigarettes a superior cessation tool	• *"With e-cigarettes you still get to blow out smoke. Because I tried using that Nicorette inhaler, and it just …Ugh..It just didn't stick with me. I guess because I'm not blowing out smoke, so it felt like I'm not doing anything. And the nicotine gum, that stuff kind of bites the back of your throat, and I don't really like that feeling."*- Male, 26 years old (35)
	3.2 E-cigarettes easy to obtain and use	• *"I actually wanted to get it because it was cheaper than smoking cigarettes, because I was smoking like a pack, a pack and a half per day. So it was a lot cheaper than purchasing the cigarettes, which were like $8, if you're lucky. I mean every pack."* - Male, 25 years old *(35)*
	3.3 A novel product which appeals to young people	• *"I can do it while studying in the library, upstairs and no one would really know."*- Demographic not recorded (30)
	3.4 E-cigarette companies aggressively targeting young adults	• *"I think the more ads they put up, the more inclined younger people are to try it. Especially if they are flavoured, it'd be interesting to try them."*- Female 17 years old (58)<br>• *"The same rules should apply because they're the same thing aren't they. They've both got nicotine in them."* - Male, 17 years old, male, e-cigarette user and traditional cigarette user (31)
	3.5 E-cigarettes perceived as a harmful and risky product	• "Nothing is really good for you when you inhale it*, doesn't matter what. But these chemicals, putting them into your lungs, it's still not good for you regardless of what it is, and I'm aware of that, but it's a self-conscious choice that everyone has to make."* - Female, 19 years old (37)
	3.6 Concern and confusion regarding e-cigarette contents and safety	• *"I don't know if they're any better for you than cigarettes because I feel like there's a lot of mystery behind them, but I hope* [they are better for you than cigarettes]. *"* - Demographic not recorded (55)
	3.7 Indifference about potential harm	• F4: *"In the shop I went to in the market, there was side effects on erm the thing, it was just like drowsiness and all that. It wasn't anything major."*<br>F1: *"So you still thought you'd buy that?"*<br>F4: *"Yeah."*<br>F1: *"Even though it said it can cause that?"*<br>F4: *"It's not anything, it's nothing major…"*<br>F1: *"Its minor, but it's still a side effect…"*<br>F4: *"If it makes you a bit sleepy than that's fine, it's not like a proper drug."*<br>- F1: Female, 16 years old, non-e-cigarette user and non-traditional cigarette user<br>- F4: Female, 16 years old, e-cigarette user and traditional cigarette user (31)
